# The italian national genomic strategy: current status, challenges, and future perspectives in clinical practice and public health

**DOI:** 10.1007/s12687-025-00828-w

**Published:** 2025-10-13

**Authors:** Francesco Andrea Causio, Sara Farina, Alessandra Maio, Flavia Beccia, Luigi Russo, Valentina Baccolini, Matteo Chiara, Americo Cicchetti, Gualtiero I. Colombo, Giovanni Comandé, Domenico Coviello, Ruggero De Maria, Massimo Delledonne, Corrado De Vito, Daniela Galeone, Paolo Gasparini, David Horner, Giovanni Martinelli, Carolina Marzuillo, Laura Palazzani, Erica Pitini, Maurizio Sanguinetti, Aldo Scarpa, Marco Tartaglia, Francesco Danilo Tiziano, Giovanni Tonon, Bruno Dallapiccola, Paolo Villari, Giovanna Elisa Calabrò, Stefania Boccia

**Affiliations:** 1 Section of Hygiene, Department of Life Sciences & Public Health, Università Cattolica del Sacro Cuore, Rome, 00168 Italy; 2 Department of Public Health and Infectious Diseases, Sapienza University of Rome, Rome, Italy; 3 Department of Biosciences, University of Milan, Milan, Italy; 4 Graduate School of Economics and Healthcare Management (ALTEMS), Faculty of Economics, Università Cattolica del Sacro Cuore, Rome, Italy; 5 Immunology and Functional Genomics Unit, Centro Cardiologico Monzino IRCCS, Milan, Italy; 6 Lider-Lab, Sant’Anna School of Advanced Studies of Pisa, Pisa, Italy; 7 Laboratory of Human Genetics, IRCCS Giannina Gaslini Institute, Genoa, Italy; 8 Institute of General Pathology, Università Cattolica del Sacro Cuore, Rome, Italy; 9 Department of Biotechnology, University of Verona, Verona, Italy; 10 Directorate-General for Health Prevention, Ministry of Health, Rome, Italy; 11 Medical Genetics, IRCCS Burlo Garofolo Maternal and Child Hospital, Trieste, Italy; 12 Scientific Institute of Romagna for the Study and Treatment of Cancer (IRST) IRCCS, Meldola, Italy; 13 Free University Maria SS. Assunta (LUMSA), Rome, Italy; 14 Department of Laboratory Sciences and Infectious Diseases, A. Gemelli, IRCCS University Hospital Foundation, Rome, Italy; 15 ARC-Net Research Center, Department of Diagnostics and Public Health, University of Verona, Verona, Italy; 16 Molecular Genetics and Functional Genomics, IRCCS, Ospedale Pediatrico Bambino Gesù, Rome, 00146 Italy; 17 Complex Unit of Medical Genetics, Fondazione Policlinico Universitario IRCCS “A. Gemelli”, Rome, Italy; 18 Cancer Functional Genomics Unit, Division of Molecular Oncology, IRCCS San Raffaele, Milan, Italy; 19 Department of Human Sciences, Society and Health, University of Cassino and Lazio Meridionale, Cassino, Italy; 20 Department of Woman and Child Health and Public Health, Fondazione Policlinico Universitario A. Gemelli IRCCS, Rome, Italy

**Keywords:** Genomic medicine, Public health genomics, Healthcare infrastructure, Precision medicine

## Abstract

This article presents the outcomes of a national initiative aimed at developing a technical document to support the future Italian National Genomic Strategy, carried out from 2021 to 2024 through the collaboration of 14 research institutions. The project was designed to align with major European genomic initiatives, particularly the “1 + Million Genomes” (1 + MG) Declaration and its supporting programs, including Beyond 1 Million Genomes (B1 + MG), the Genomic Data Infrastructure (GDI), and Genome of Europe (GoE). The initiative was structured around 12 National Mirror Groups (NMGs), each addressing a specific domain such as clinical implementation, ethical and legal issues, data governance, health economics, and public engagement. Through expert consensus and coordinated activities, the project produced a comprehensive technical document outlining seven strategic lines and related intervention areas. These include the integration of genomic testing into clinical practice, development of specialized genomic centers, creation of a national genomic data infrastructure, professional training, and public education. The proposed strategy emphasizes equitable access to genomic medicine, the use of health technology assessment to evaluate new technologies, and the importance of citizen engagement and literacy. By fostering collaboration among institutions, healthcare professionals, and the public, the final goal is to position Italy as a leader in genomic medicine and ensure the responsible, effective, and ethical use of genomics in public health and clinical care.

## Introduction

The genomic revolution has transformed healthcare systems worldwide through technological innovations and biomolecular discoveries over the last few decades, allowing for novel diagnostic and therapeutic pathways in a broad range of conditions. In Italy, this transformation began with the establishment of the first European policy dedicated to genomics in 2013 through the “Guidelines for Genomics in Public Health” (Conferenza Stato-Regioni 2013), followed in 2017 by the “Plan for Healthcare System Innovation based on Omics Sciences” (Boccia et al. 2017) and in 2022 by “The Priorities of the National Genomics Plan” (Locatelli et al. 2022). In 2018, Italy joined twelve other EU Member States by signing the “*Towards access to at least 1 million sequenced genomes in the EU by 2022*” (1 + MG) declaration (European Commission, 2023). This initiative aimed to improve European competitiveness in personalized medicine by developing predictive, preventive, and participatory healthcare approaches. This initiative expanded to include 27 Countries and was supported by the European Commission’s DG Connect co-funding three specific projects: Beyond 1 Million Genomes (B1 + MG, https://b1mg-project.eu/), the Genomic Data Infrastructure (GDI, https://gdi.onemilliongenomes.eu/) and more recently the Genome of Europe (GoE, https://digital-strategy.ec.europa.eu/en/news/genome-europe-project-launched-first-step-towards-european-reference-genome) projects. These EU-funded initiatives aim at creating a European network for cross-border access to genomic and clinical data, facilitating cross-country data exchange and benefitting patients, practitioners, and researchers alike. In Italy, the implementation of these initiatives was accompanied by the “*Italian Genomic Strategy: Establishment of a Steering Committee to support the European initiative 1 + Million Genomes (1 + MG) and Beyond 1 + MG (B1 + MG)*,* and the Inter-institutional Coordination for Genomics in Public Health*” project funded by the Center for Disease Control (CCM) of the Italian Ministry of Health (MoH), aiming at developing technical documents in Public Health Genomics to support future institutional programs and actions. The funding of this project by the Italian Ministry of Health underscores the national institutional commitment to the field of genomics and its implementation, in line with the recognized European-level action priorities.

## Methods

The CCM project ran for 30 months, from December 2021 to June 2024, as a multi-institutional collaboration between 14 Italian research centers and universities. Each partner institution was responsible for specific technical aspects required for the implementation of genomic-based applications in public health. The project partners, listed as authors in this manuscript, were divided into 12 different National Mirror Groups (NMGs), each working in parallel with their European counterparts of the 1 + MG initiative Working Groups (WGs), previously established by the time the CCM project had started. The NMGs were as follows: NMG1: Scope stakeholders and governance, NMG2: Ethical Legal and Societal Issues (ELSI), NMG3: Common standards for capturing clinical and phenotypic data requirements, NMG4: Good sequencing practice/development of standards for clinical interpretation, NMG5: Interoperability and data access governance, NMG6: Health economics and outcome research, NMG7: Involvement of the private sector, NMG8: Use Case - Rare Diseases, NMG9: Use Case - Cancer, NMG10: Use Case - Common complex diseases, Populations, Precision prevention, Pharmacogenomics, NMG11: Use Case - Infectious diseases, focused on genomic characterization of host susceptibility factors and personalized therapeutic approaches for infection control, NMG12: Genome of Europe.

The methodological approach involved three main phases. In the first, a comprehensive analysis of existing genomic initiatives and facilities in Italy was performed. This analysis served the purpose of identifying the most relevant centers and initiatives, while providing an overview of the system’s readiness to public health genomics. Secondly, the experts involved in the project developed technical recommendations through expert consensus, aiming at identifying the most important priorities for fostering Public Health Genomics in Italy. Finally, these findings coalesced into a unified strategic document that forms the backbone of the Italian National Genomic strategy.

## Results

The project partners served as a steering committee to support the Ministry of Health in harmonizing inputs from the European 1 + MG initiative as well as B1 + MG at the national level. Moreover, the project provided a technical-scientific document to support, based on available scientific evidence and the opinion of national experts, the MoH decision-makers and policy-makers in implementing Public Health Genomics in the National Health Service, aligning with the European 1 + MG objectives and activities. The document is structured into seven interconnected Strategic Lines, addressed by specific Intervention Areas (Table 1), spanning from the clinical implementation of genomic testing for rare diseases, oncology, and complex disorders to the development of specialized genomic centers and networks with integrated IT infrastructure for data management; professional training of healthcare providers in genomic medicine; support for basic and translational research; and public engagement through awareness campaigns and genomic literacy programs, also implementing the informed consent. This integrated approach aims to position Italy at the forefront of genomic medicine while ensuring standardized implementation across the national territory, focusing on equitable access to advanced genomic technologies and personalized medicine approaches.

**Table 1:** Table of contents – Strategic lines and intervention areas of the strategic document that informs the Italian national Genomic Strategy

Strategic Line	Intervention Areas
Strategic Line 1: Clinical Practice and Public Health	1. Prevention: Develop Predictive and Preventive Medicine to improve Public Health and Well-Being 1a. Prenatal Screening 1b. Neonatal Screening 1c. Adult Screening 1c.1. Oncological Screening 1c.2. Screening for Common Diseases 2. Diagnosis and Personalized Medicine 2a. Rare Diseases and Undiagnosed Patients 2b. Oncological Diseases 2c. Complex Diseases and Pharmacogenetics 2d. Infectious Diseases
Strategic Line 2: Organizational-Healthcare Model	1.Development and Implementation of Specialized Clinical-Care Networks: 1a. Rare Diseases 1b. Oncological Diseases
Strategic Line 3: Infrastructure and Data Management	1. Development of an Ethical-Legal Infrastructure for the Primary and Secondary use of Genomic Data 2. Implementation of Advanced IT Infrastructure for Genomic Data Storage 3. Integration of Digital Tools for Genomic Data Management 4. Creation and Management of a National Genome Database 5. Cybersecurity Unit for Genomic Data Protection 6. Data Engineering Unit for maintaining the National Database 7. National Contact Point for the European Health Data Space
Strategic Line 4: Research and Innovation	1. Basic and Translational Research with a Research-Clinical Interface 2. Health Technology Assessment (HTA) 3. Ethical-Legal Guidelines for Genomic Research 4. Advanced Technologies for analyzing Large Genomic Datasets 5. Industry Collaborations for Sustainable Innovation 6. International Cooperation for Precision Health
Strategic Line 5: Capacity Building for Healthcare Professionals	1. Recruitment of healthcare professionals 2. Training and Continuous Professional Development 3. Awareness and Information Campaigns
Strategic Line 6: Engagement of Citizens and Patients	1. Engagement Strategies 2. Health Education and Genomic Literacy 3. Actions to Build Population Trust
Strategic Line 7: Institutional Governance and Stakeholders	1. Architecture and Governance of the National Health System (SSN) Network for Genomics 2. Stakeholder Involvement 3. International Partnerships (public and private)

The proposed strategy emphasizes the importance of establishing a clear procedural pathway and a rigorous methodology to evaluate the effectiveness and costs of these technologies, as well as their potential short- and long-term benefits for patients and their families, through the Health Technology Assessment process. In addition, it supports the consolidation of continuous education on the potential applications of genomics in clinical sciences for healthcare professionals through mandatory, credit-bearing programs. While existing regional initiatives have shown success, the proposed strategy calls for more cohesive nationwide training delivered through structured formats, including distance learning. Additionally, it advocates for broadening public engagement through innovative solutions, such as a national genomics day and web portal to share guidelines and research. It also aims to educate people about genomics medicine’s impacts, especially regarding consumer genetic testing. It could be expected that implementation will occur over 1–2 years through partnerships between health authorities, universities, and scientific organizations, supported by dedicated funding.

## Discussion

Implementing a National Genomic Strategy is crucial in advancing personalized medicine and public health at a national healthcare system level. In Italy, this effort builds on a discrete record of available norms in personalized medicine (Causio et al. 2022). This is particularly true in a country like Italy, where the health governance is devolved to 21 Regions and Autonomous Provinces responsible for the organization and delivery of health services. Integrating genomic analysis into clinical practice shows significant potential, particularly in rare diseases where 60–70% of diagnostic yields can be achieved through exome sequencing (Dallapiccola 2024), potentially shortening the 4-year “diagnostic odyssey” many rare disease patients face (Dallapiccola 2024). Moreover, to foster their implementation in clinical practice, it is essential to develop a standardized evaluation framework tailored to the context of the Italian NHS that can address the methodological challenges these technologies present. The transition toward precision medicine through molecular tumor profiling in oncology represents another promising avenue. Italy lags behind other countries in clinical trial participation rates for oncology, leaving room for improvement in translational research implementation. (Lappalainen et al. 2015) Developing population-specific genomic databases, mainly through initiatives like GoE, presents a significant opportunity to improve variant interpretation and risk assessment. Italy’s contribution is crucial to creating a comprehensive reference database reflecting the European population’s genetic diversity to implement accurate polygenic risk scores (PRS) for complex diseases. The creation of federated networks for genomic data sharing, as outlined in the B1 + MG project recommendations, will require significant investment but is essential for supporting international genomic medicine initiatives. The legal framework for data sharing and protection needs careful consideration, particularly regarding implementing the General Data Protection Regulation (GDPR) requirements while facilitating research access. The proposed strategy emphasizes the need for structured training programs and the creation of specialized centers of excellence working in hub-and-spoke networks. This organizational model could help address current disparities in access to genomic medicine across different regions of Italy.

A challenge lies in increasing genomic literacy among the general population, particularly given the rapid advancement of genomic technologies and their increasing accessibility through direct-to-consumer testing. The proposed strategy recognizes three essential components for effective genomic medicine implementation: public literacy, engagement, and capacity building. Citizens, both healthy and ill, must be empowered to make informed decisions - through ethically designed informed consent - about their health and understand the implications of sharing personal genomic data with trusted institutions. Recent reports found that Italian citizens have positive attitudes and a desire to deepen their knowledge of genetic testing and personalized medicine (Calabrò et al. 2024; Causio et al. 2024). Integrating genomic screening programs, particularly in prenatal and neonatal settings, shows promise for early intervention and prevention (Stark and Scott 2023). However, as highlighted in the recommendations for implementation, careful consideration must be given to ethical implications and the development of clear clinical pathways for managing results. Additionally, the decentralized Italian NHS governance could slow down or impair the implementation of Public Health Genomics. To this purpose, national policy-makers should make sure to include regional decision-makers and interested parties in the relevant settings, to ensure all voices are considered. Doing so, harmonized progress can be ensured, and citizens will benefit at a national level.

Our work showcases the efforts of Italy to pave the way for implementing Public Health Genomics in its healthcare system, considering potential pitfalls that could limit the benefits. Of course, further investments will be necessary to implement the actions outlined in this proposal.

As demonstrated by European experiences, adopting Public Health Genomics requires investments in sequencing technologies and, primarily, the ability to reshape the entire healthcare system through multilevel governance. While far from over, the ongoing efforts will require the plural involvement of all voices that represent the different stakeholders in the healthcare system. A participatory model is crucial, involving all stakeholders - from institutions to healthcare professionals, from industry to citizens - in a shared decision-making process that aligns healthcare policies, professional training, and technological infrastructure with population needs.

## Data Availability

No datasets were generated or analysed during the current study.
